# Childbirth dynamics in the riverside region of the Brazilian Amazon from the perspective of geospatialization

**DOI:** 10.1590/0034-7167-2024-0038

**Published:** 2025-01-10

**Authors:** Liandra Silva Lopes, Laura Maria Vidal Nogueira, Erlon Gabriel Rego de Andrade, Ivaneide Leal Ataíde Rodrigues, Juan Andrade Guedes, Rosinelle Janayna Coêlho Caldas, Paula Gisely Costa Silva

**Affiliations:** I Universidade Federal do Pará. Belém, Pará, Brazil; II Universidade do Estado do Pará. Belém, Pará, Brazil

**Keywords:** Spatial Analysis, Epidemiology, Obstetrics, Parturition, Public Health, Análisis Espacial, Epidemiología, Obstetricia, Parto, Salud Pública

## Abstract

**Objective:**

to analyze the spatial-temporal pattern of childbirths and flow of postpartum women assisted at a regional reference maternity hospital.

**Methods:**

ecological study of 4,081 childbirths, between September 2018 and December 2021, at a public maternity hospital in the Baixo Tocantins region, Pará, Brazil. With data collected from five sources, a geographic database was constructed, and spatial analysis was used with Kernel density interpolator. Maps were generated using *QGis*/3.5 and *TerraView*/4.3, calculating chi-square (p<0.05).

**Results:**

the highest concentrations of normal and cesarean childbirths were observed in Barcarena (n=2,558/62.68%), Abaetetuba (n=750/18.38%), Moju (n=363/8.89%) and Igarapé-Miri (n=219/5.37%). Among the municipalities in the region, ten had obstetric beds, totaling 210 beds. In this scenario, postpartum women traveled up to 288 km to reach the maternity hospital.

**Conclusions:**

long distances between certain municipalities of residence and maternity hospital, and low supply of obstetric beds, were identified as risk factors for unfavorable obstetric outcomes.

## RESUMEN

**Objetivo:**

analizar el patrón espacio-temporal de los nacimientos y el flujo de parturientas atendidas en una maternidad de referencia regional.

**Métodos:**

estudio ecológico de 4.081 nacimientos realizados de septiembre/2018 a diciembre/2021 en una maternidad pública de la región de Baixo Tocantins, Pará, Brasil. Con datos recolectados de cinco fuentes, se construyó una base de datos geográficos y se utilizó análisis espacial con el interpolador de densidad Kernel. Los mapas se generaron utilizando los *softwares QGis*/3.5 y *TerraView*/4.3, calculando el chi-cuadrado (p<0,05).

**Resultados:**

las mayores concentraciones de nacimientos, por vía normal y por cesárea, se evidenciaron en Barcarena (n=2.558/62,68%), Abaetetuba (n=750/18,38%), Moju (n=363/8,89%) e Igarapé-Miri (n=219/5,37%). Entre los municipios de la región, diez contaban con camas obstétricas, totalizando 210 camas. En este escenario, las parturientas recorrieron hasta 288 kilómetros para llegar a la sala de maternidad.

**Conclusiones:**

las largas distancias entre ciertos municipios de residencia y la maternidad, y la baja oferta de camas obstétricas, fueron identificadas como factores de riesgo para resultados obstétricos desfavorables.

## INTRODUCTION

Among the 17 Sustainable Development Goals (SDGs), the third refers to the need to ensure healthy lives for populations and promote well-being for all, incorporating the goal of reducing the maternal mortality rate and ending preventable deaths, including newborn deaths. This is a global pact, with targets set for the year 2030, raising the various local and regional challenges that imply in achieving the goals^(1)^.

In the specific field of childbirth care, many efforts are being made nationally and internationally, with innovation in public policies, aiming to meet the SDGs. In 2018, an important strategy was developed by the United Nations Children’s Fund (UNICEF), called the Every Child Alive Campaign, which encourages the prevention and timely treatment of complications arising from childbirth, establishing two priorities: the qualification of healthcare professionals; and the guarantee of necessary supplies, in all institutions that provide obstetric care, to provide safe and effective care^(2,3)^.

In this context, Brazil has presented several initiatives to achieve, by 2030, the goal of preserving women’s health and integrity during childbirth and not exceeding 70 maternal deaths per 100,000 live births^(4)^. Among them, the initiative of the Brazilian Health System (SUS - *Sistema Único de Saúde*) Brazilian National Commission for the Incorporation of Technologies (CONITEC - *Comissão Nacional de Incorporação de Tecnologias*) stands out, which, in 2017, published the Brazilian National Guidelines for Normal Birth Care, with the aim of standardizing obstetric practices based on scientific evidence, reducing the number of unnecessary interventions and, thus, contributing to promoting more qualified care to women^(5)^.

Also noteworthy is the implementation of Healthcare Networks, such as the Stork Network, created in 2011^(6)^, and the Personalized Childbirth Project, instituted in 2015^(7)^. It is important to note that, from conception to the puerperium, women’s rights are guaranteed by Ordinance 1.067 of July 4, 2005, which established the Brazilian National Policy for Obstetric and Neonatal Care, guiding the health departments of municipalities, states and the Federal District to develop actions to promote health, prevent illness and provide care to pregnant women and newborns^(8)^.

Such initiatives aim to reduce maternal mortality, which, in 2022, in Brazil, reached figures of 66,862 deaths in the age group of 10 to 49 years. In turn, in the state of Pará, considering this age group and the same year, there were 2,873 deaths^(9)^. Among the factors related to high mortality rates are complications during childbirth.

In this context, it is worth noting that the COVID-19 pandemic has introduced additional challenges in the quest to reduce maternal mortality. Initially, pregnant and postpartum women were not included as groups most vulnerable to severe complications from COVID-19, delaying the start of vaccination against the disease in these groups. The repercussions of the pandemic were profound in the SUS, contributing to unfavorable perinatal outcomes, such as increased maternal deaths from indirect and preventable obstetric causes^(10)^.

Events such as complications and deaths do not occur uniformly throughout the territory, and it is important to study regional differences in order to adopt targeted strategic actions. Despite the legitimacy of public policies, many challenges remain in fully implementing them, given the adversities that arise in the daily provision of healthcare and the management of public services. Among the challenges, the heterogeneous composition of the Brazilian population stands out, with social groups exhibiting different mortality rates, in addition to the geographic characteristics of the territory, which, in many situations, compromise access to services and qualified care, as is the case in the riverside municipalities of the Amazon in Pará^(11)^.

Located on the banks of rivers and streams, these municipalities face a lack of specialized healthcare services, maternity wards and medical transport that ensure women have safe access to the health unit for prenatal consultations and to the place of childbirth, making an important recommendation of the Stork Network unfeasible. Thus, there is a contrast between public policies and their real applicability, especially for riverside women in the Amazon region of Pará^(12,13)^.

Still in the context of policies to assist women during childbirth, it is worth highlighting the strategy of agreeing on services between municipalities, provided as a model in the SUS, through the Organizational Contract for Public Health Action (COAP - *Contrato Organizativo de Ação Pública da Saúde*), which guides the organization and integration of health actions and services in Brazilian regions, aiming at comprehensive care and emphasizing managers’ responsibilities^(14)^.

Thus, intermunicipal agreements have been adopted in health management; however, its applicability and suitability to the various Brazilian realities must be questioned, as in the case of the Baixo Tocantins region, one of the integration regions of the state of Pará, which has a reference maternity hospital to assist the 11 municipalities that comprise it. Since it is a region with a riverside population, peculiar situations arise, and this model of agreement on services among municipalities should be assessed and monitored regularly.

## OBJECTIVE

To analyze the spatial-temporal pattern of childbirths and flow of postpartum women assisted at a regional reference maternity hospital.

## METHODS

### Ethical aspects

The study complied with Resolution 466/2012 of the Brazilian National Health Council/Ministry of Health, and institutional authorization was obtained from the Pará State Department of Public Health (SESPA - *Secretaria de Estado de Saúde Pública do Pará*) and from a reference maternity hospital linked to the SUS, with approval by the *Universidade do Estado do Pará* Undergraduate Course in Nursing Research Ethics Committee. Since this was a study with secondary data, Database Access Authorization Terms were used, which were signed by representatives of SESPA and the maternity hospital, which is why participants’ consent was waived.

### Study design and location

An ecological study was chosen, using geotechnology tools applied to health. Its writing was guided by STrengthening the Reporting of OBservational studies in Epidemiology (STROBE)^(15)^.

The study took place in the Baixo Tocantins region, state of Pará, in the Brazilian Amazon, which has 839,022 inhabitants^(16)^, distributed across 11 municipalities: Abaetetuba, Acará, Baião, Barcarena, Cametá, Igarapé-Miri, Limoeiro do Ajuru, Mocajuba, Moju, Oeiras do Pará and Tailândia^(17)^. The public maternity hospital, the setting for this study, is located in the municipality of Barcarena and receives demand from all municipalities in the region.

### Period, population and selection criteria

The maternity hospital began its activities in September 2018 and, in 2022, received the Baby-Friendly Hospital certification from the Ministry of Health, recognizing its good practices, humanized care and breastfeeding encouragement^(18)^. In view of this, this study considered the period from September 2018 to December 2021, in which 4,081 childbirths were performed, corresponding to the total number included in this study. The records of childbirths whose postpartum women resided in the municipalities of the Baixo Tocantins region were included, without excluding cases.

### Study protocol

The data were obtained in August 2022, from five sources: 1) Live Birth Information System (SINASC - *Sistema de Informação sobre Nascidos Vivos*), whose data were obtained from SESPA and refer to the number of live births in the maternity ward, corresponding to women in the Baixo Tocantins region; 2) Hospital Information System (SIH-SUS - *Sistema de Informações Hospitalares*), with data obtained from the Barcarena Municipal Health Department (SEMUSB - *Secretaria Municipal de Saúde de Barcarena*), extracting the variables such as year of childbirth notification, mother’s age, color/race, education in years, marital status, duration of pregnancy, mode of delivery and place of residence; 3) specific maternity database, from which additional information was extracted on the mode of travel of postpartum women between their home and maternity ward, as well as whether there was a referral from another service or whether the search for the maternity ward was spontaneous; 4) Brazilian National Registry of Health Establishments (CNES - *Cadastro Nacional de Estabelecimentos de Saúde*), through the SUS Information Technology Department (DATASUS - *Departamento de Informática do SUS*), to consult the number of obstetric beds in the municipalities; 5) virtual environment of the Brazilian Institute of Geography and Statistics (IBGE - *Instituto Brasileiro de Geografia e Estatística*), with public access, in order to obtain demographic variables (population quantity of the municipalities) and cartographic variables (latitude and longitude of the municipalities) to build the geoinformation layers of the municipal boundaries.

### Analysis of results, and statistics

The database was debugged in order to organize information and exclude unnecessary attributes for the study, using Microsoft Office Excel, version 2019, and *TabWin*/Ministry of Health. The nonparametric Chi-square statistical test of equal expected proportions (adherence test for uniform probability models) was applied, with p<0.05, to analyze data related to mother’s age, color/race, education, marital status, duration of pregnancy and mode of delivery.

The geographic database (BDGeo) was constructed and then spatial analysis was performed using the Kernel density interpolator, which generated the visual expression of childbirth density. Thematic maps, with the distribution of cases and the childbirth indicator of the municipalities, were generated using *QGis*, version 3.5, and *TerraView*, version 4.3.

## RESULTS

**Figure 1:**
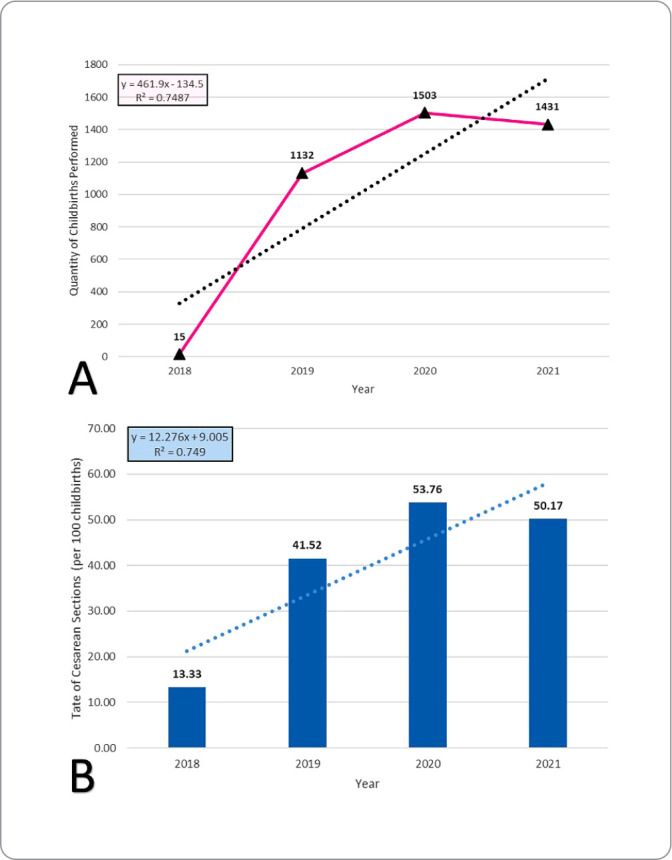
Trend in demand and rate of cesarean sections for postpartum women treated at the regional maternity hospital, from September 2018 to December 2021. Pará, Brazil, 2022

A total of 4,081 childbirth records were analyzed, of which 15 (0.37%) occurred in 2018; 1,132 (27.74%), in 2019; 1,503 (36.83%), in 2020; and 1,431 (35.06%), in 2021, as shown in Figure 1A. Concerning cesarean sections, in 2018, the rate was 13.33 per 100 childbirths, and in the following years, this indicator increased, with a linear growth trend (Figure 1B).

In relation to the social profile of postpartum women, the age group of 19 to 28 years had the highest proportion (n=2,304; 56.46%), however there was a high number of childbirths among adolescents (n=714; 17.50%), considering the Statute of Children and Adolescents classification in Brazil^(19)^, which defines adolescence as the period between 12 and 18 years of age. It was identified that 2,716 (66.55%) were brown and 1,252 (30.68%) had no record. The most expressive length of education was eight to 11 years (n=2,362; 57.88%), followed by four to seven years (n=1,059; 25.95%). Regarding marital status, 2,000 (49.01%) women lived in a stable union and 1,192 (29.21%) were single (Table 1).

**Table 1:** Social profile and duration of pregnancy of postpartum women assisted at the regional maternity hospital, from September 2018 to December 2021. Pará, Brazil, 2022 (N=4,081)

Variables	n	%	*p* value
Age group			<0.0001
12 to 18 years	714	17.50	
19 to 28 years	2,304	56.46	
29 to 38 years	957	23.45	
39 to 48 years	106	2.60	
Color/race			<0.0001
Brown	2,716	66.55	
White	72	1.76	
Yellow	22	0.54	
Black	19	0.47	
Ignored	1,252	30.68	
Education			<0.0001
None	9	0.22	
1 to 3 years	147	3.60	
4 to 7 years	1,059	25.95	
8 to 11 years	2,362	57.88	
12 years or more	402	9.85	
Ignored	102	2.50	
Marital status			<0.0001
Single	1,192	29.21	
Married	816	20.00	
Stable union	2,000	49.01	
Widow	4	0.10	
Legally separated	12	0.29	
Ignored	57	1.40	
Duration of pregnancy			<0.0001
Less than 22 weeks	71	1.74	
22 to 27 weeks	341	8.36	
28 to 31 weeks	1,302	31.90	
32 to 36 weeks	2,326	57.00	
37 to 41 weeks	41	1.00	

As for duration of pregnancy, the most significant values were 32 to 36 (n=2,326; 57.00%) and 28 to 31 weeks (n=1,302; 31.90%). It is worth noting that 71 (1.74%) presented gestational evolution of less than 22 weeks and that only 41 (1.00%) had 37 to 41 weeks (Table 1).

According to Table 2, the occurrences of vaginal and cesarean delivery presented, respectively, higher proportions among postpartum women in the municipalities of Barcarena (n=1,366/65.58%; n=1,192/59.66%) and Abaetetuba (n=347/16.66%; n=403/20.17%). In this context, the municipalities of Limoeiro do Ajuru (n=4/0.19%; n=8/0.40%) and Tailândia (n=3/0.14%; n=7/0.35%) presented the lowest records.

**Table 2:** Mode of delivery, according to municipality of residence, of postpartum women assisted at the regional maternity hospital, from September 2018 to December 2021. Pará, Brazil, 2022 (N=4,081)

Municipality/year	Mode of delivery						
	**Normal**						
	**2018**	**2019**	**2020**	**2021**	**Total**	**%**	***p* value**
Abaetetuba	1	157	101	88	347	16.66	<0.0001
Acará	0	22	11	9	42	2.02	
Baião	0	7	11	5	23	1.10	
Barcarena	12	378	478	498	1,366	65.58	
Cametá	0	3	2	3	8	0.38	
Igarapé-Miri	0	29	34	42	105	5.04	
Limoeiro do Ajuru	0	0	2	2	4	0.19	
Mocajuba	0	2	4	5	11	0.53	
Moju	0	59	49	55	163	7.83	
Oeiras do Pará	0	5	2	4	11	0.53	
Tailândia	0	0	1	2	3	0.14	
Total	13	662	695	713	2,083	100.00	
	**Cesarean section**						
	**2018**	**2019**	**2020**	**2021**	**Total**	**%**	***p* value**
Abaetetuba	0	126	166	111	403	20.17	<0.0001
Acará	0	0	8	8	16	0.80	
Baião	0	6	8	9	23	1.15	
Barcarena	2	251	490	449	1,192	59.66	
Cametá	0	6	1	3	10	0.50	
Igarapé-Miri	0	22	44	48	114	5.71	
Limoeiro do Ajuru	0	4	2	2	8	0.40	
Mocajuba	0	1	6	9	16	0.80	
Moju	0	52	78	70	200	10.01	
Oeiras do Pará	0	2	4	3	9	0.45	
Tailândia	0	0	1	6	7	0.35	
Total	2	470	808	718	1,998	100.00	

**Figure 2:**
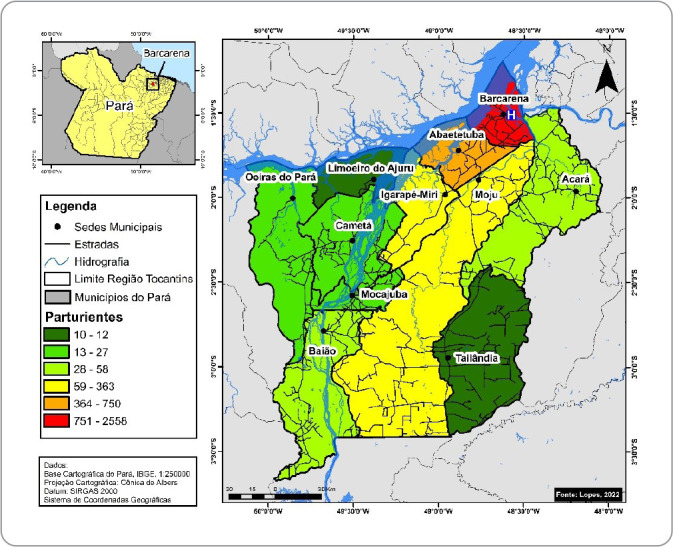
Spatial distribution of childbirths performed in the regional maternity hospital, from September 2018 to December 2021. Pará, Brazil, 2022

Considering the sum of childbirths by normal and cesarean sections, Figure 2 illustrates the different concentrations among the municipalities. Thus, the highest concentrations were evidenced in Barcarena (n=2,558; 62.68%), Abaetetuba (n=750; 18.38%), Moju (n=363; 8.89%) and Igarapé-Miri (n=219; 5.37%), and the lowest concentrations were recorded in the other municipalities: Acará (n=58; 1.42%), Baião (n=46; 1.13%), Mocajuba (n=27; 0.66%), Oeiras do Pará (n=20; 0.49%), Cametá (n=18; 0.44%), Limoeiro do Ajuru (n=12; 0.29%) and Tailândia (n=10; 0.25%).

**Figure 3:**
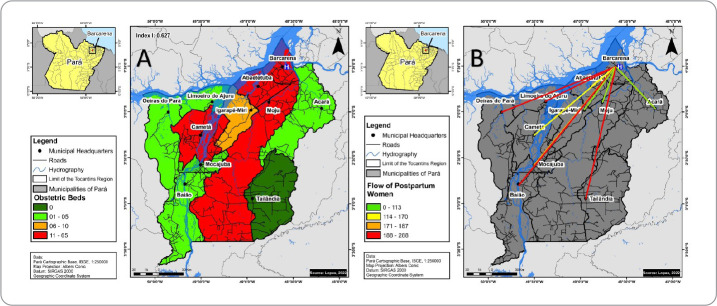
Spatial distribution of obstetric beds and flow of postpartum women assisted at the regional maternity hospital, from September 2018 to December 2021. Pará, Brazil, 2022

According to Figure 3A, among the 11 municipalities that make up the Baixo Tocantins region, ten had obstetric beds reported in CNES, totaling 210 beds, distributed as follows: Barcarena (65 beds), Abaetetuba (60 beds), Cametá (42 beds), Moju (18 beds), Igarapé-Miri (10 beds), Limoeiro do Ajuru (five beds), Baião (three beds), Oeiras do Pará (three beds), Acará (two beds) and Mocajuba (two beds).

In turn, Figure 3B contemplates the mobility of postpartum women seeking hospital care to give birth, with flows of up to 288 km between the municipality of residence and the regional maternity hospital. The municipalities of Baião, Oeiras do Pará and Tailândia are the furthest away from the maternity hospital, a scenario in which access from Oeiras do Pará to Barcarena, where this institution is located, is exclusively by river, given the existence of rivers that crisscross the territory. Although they are relatively close to Barcarena, the municipalities of Limoeiro do Ajuru and Cametá are separated by rivers, representing a significant obstacle to travel.

## DISCUSSION

The spatial pattern of childbirths indicates a predominance of women from the municipality where the regional maternity hospital is located and from municipalities that are geographically closer. However, it is possible to see a significant flow throughout the Baixo Tocantins region.

It is a fact that the demand for childbirths in this maternity hospital is also related to the population and geographic distance, which implies ease or difficulty of access in terms of travel logistics. Thus, the greater proportion evidenced in the municipality of Barcarena is due to the fact that it is the headquarters of the maternity hospital, and in Abaetetuba, the proportion is explained by it being a neighboring municipality, with greater ease of intercity travel.

Care for women during childbirth showed an upward trend during the period studied, and cesarean sections followed this trend. It is understood that, because it was the first reference maternity hospital in the region, women began to choose it or were referred due to the possible provision of more qualified care, or even due to the shortage of obstetric beds in the various municipalities, in addition to the expectation of being better assisted.

It is understood that the growing demand is linked to service implementation dissemination, promoting spontaneous search by women and referral by health teams in the municipalities of the region. The characteristic of this institution as a reference maternity hospital implies the reception of more complex cases, which require differentiated care, such as cases that require cesarean section surgery^(20,21)^. Even so, the hospital’s policy values the viability of natural childbirth, whenever indicated, resulting in a reduction in the rate of cesarean sections in 2021 when compared to 2020.

The performance of cesarean sections without proper indication has been the subject of debate and research, with the aim of reducing them, considering that the rates of this type of surgery have increased globally, corresponding to more than one in every five childbirths (21.00%), according to data from the World Health Organization (WHO) and the Pan American Health Organization (PAHO). In Latin America, cesarean sections account for four out of every ten childbirths, and in Brazil, they have already surpassed the number of normal childbirths, despite the fact that access to the surgery is unequal among women^(22)^.

Similar findings were obtained in a national study that investigated the types of childbirth in the SUS, identifying an increase in cesarean sections in all regions of the country in recent years. However, the North region stood out for the predominance of normal childbirths, but has shown an exponential growth in cesarean sections, indicating that it may follow the rate of other regions of the Brazilian territory in the coming years^(23)^. In the context of health insurances in Brazil, this growth trend is significant, given that, in 2019, of the 287,166 childbirths performed, 84.76% were by cesarean section^(24)^.

Although cesarean section surgery requires a plausible clinical indication to be performed, the current Brazilian scenario suggests an increase in the number of unnecessary and potentially harmful procedures for the mother/child dyad^(25)^. However, it should be noted that the regional maternity hospital in Barcarena assists, in addition to normal-risk postpartum women, high-risk ones, which justifies the number of cesarean sections performed there^(26)^.

Regarding the social profile of postpartum women, the prevalent age range follows the national average, aged between 19 and 28 years^(23)^. However, the region is a site of adolescent pregnancies, requiring bolder intersectoral policies. To reduce the incidence of early and/or unwanted pregnancies, the strategy for Improvement and Innovation in Care and Education in Obstetrics and Neonatology (APICE ON - *Aprimoramento e Inovação no Cuidado e Ensino em Obstetrícia e Neonatologia*) stands out, proposed by the Ministry of Health, in partnership with the Brazilian Company of Hospital Services (EBSERH - *Empresa Brasileira de Serviços Hospitalares*), the Brazilian Association of University Teaching Hospitals (ABRAHUE - *Associação Brasileira de Hospitais Universitários de Ensino*), the Brazilian Ministry of Education (MEC - *Ministério da Educação*), the Fernandes Figueira Institute (IFF - *Instituto Fernandes Figueira*) and the Oswaldo Cruz Foundation (FIOCRUZ - *Fundação Oswaldo Cruz*). Among other purposes, this strategy aims to achieve best practices in postpartum and post-abortion reproductive planning and in care for women who are victims of sexual violence and/or who have had an abortion or have undergone a legal abortion^(27)^.

In Brazil, in 2020, 53 out of every 1,000 adolescents became pregnant early, according to the State of World Population Report, published by the United Nations Population Fund (UNFPA), indicating that, in the country, this figure is above the global indicator, which is 41 cases per 1,000 adolescents. In this way, UNFPA reinforces the importance of sexual and reproductive health and of encouraging it from adolescence, in all socio-educational contexts, in addition to access to reliable information^(28)^.

Still on the profile of postpartum women, the predominance of brown women reflects the country’s ethnic scenario, whose national study of population bases, relating to the period from 1930 to 2018, identified 61.54% of brown postpartum women^(29)^. In the Brazilian population, the prevalence of the brown and black races is the result of miscegenation among Indians, Africans and other ethnic groups that participated in the country colonization, which has the greatest miscegenation in the world, due to its heterogeneous descent^(30)^.

Education, with a training period equal to or greater than eight years, represents a positive aspect, as it implies the possibility of reducing risk factors during pregnancy, childbirth and the postpartum period. This understanding is based on the fact that education of less than eight years limits, in different ways, the satisfactory development of prenatal care. Moreover, low maternal education is an eligibility criterion for the classification of newborns as at risk at birth^(31)^.

The Continuous Brazilian National Household Sample Survey (PNAD - *Pesquisa Nacional por Amostra de Domicílios Contínua*/IBGE), in 2021, showed that the average number of years of schooling for the Brazilian population aged 18 to 29, from 2012 to 2020, was 11.4 years. In this context, it is interesting to note that Brazil shows a trend of eight or more years of education, however the North and Northeast regions still have the lowest average years of education in the country, with 11.2 and 11.1 years, respectively^(32)^. The increase in the number of years studied by the Brazilian population became possible with the reduction of inequalities between human groups, although there are still many social inequalities in the country^(33)^.

Concerning marital status, the majority lived in a stable union, similar to the results of another study, which demonstrated a national average of 51.20% of women living with a partner at the time of childbirth^(23)^. This differs from the findings of research carried out in the city of Rio de Janeiro, in which single mothers accounted for 62.60%^(29)^.

Most women did not complete the ideal gestation period, as they had a gestational age of less than 37 weeks. It is known that prematurity is one of the main causes of death in the neonatal period (first 28 days of life after childbirth) and, in this study, the period in which premature childbirths were most evident was from 32 to 36 weeks, following the national average of 79.00% of premature childbirths^(34)^. Thus, considering approximately 3,000,000 childbirths per year, throughout Brazil, 2,370,000 occur with a gestational age of less than 37 weeks^(35)^.

Considering women in labor in the social and economic contexts of the region, differences in the Municipal Human Development Index (MHDI) and *per capita* income can be seen among municipalities, which have an impact on quality of life and exposure to maternal risks. The MHDI comprises three important indicators of human development: longevity, education and income. Therefore, the level measured expresses the social and financial conditions for birth and growth^(36)^.

In the scenario studied, Barcarena has the highest MHDI (0.662), followed by Abaetetuba and Baião, with 0.628 and 0.578, respectively. In turn, the lowest MHDI corresponds to the municipality of Acará (0.506). It is also worth noting that Barcarena has the highest *per capita* income in *reais* (R$ 47,684.37), Brazilian currency, followed by Acará and Limoeiro do Ajuru, with R$ 11,892.64 and R$ 14,480.24, respectively. The municipality with the lowest income is Cametá, with R$ 7,271.26^(36)^.

The MHDI’s influence was evidenced in a study carried out in the Serra Catarinense region, Brazil, whose results indicated an association with better living conditions for newborns and higher maternal education, supporting the influence of socioeconomic factors on maternal and neonatal health and living conditions^(37)^. Still in this perspective, regional studies have shown that lower MHDI values are associated with a higher incidence of adolescent pregnancy^(38,39)^.

Municipalities with lower *per capita* incomes had lower availability of obstetric beds, with the exception of the municipality of Cametá. In Brazil, the supply of hospital beds is 2.3 per 1,000 inhabitants, lower than the global average, 3.2 per 1,000 inhabitants, and the number previously recommended by the Ministry of Health, 2.5 to 3.0 per 1,000 inhabitants^(40)^. Hence, it is important to infer that social inequalities, evidenced by low *per capita* income, increase the risks of early pregnancy and negatively impact the preand post-natal periods.

In accordance with the SUS recommendations, it is worth noting that the maternity hospital meets the demands of all municipalities in the region and, despite ensuring the Stork Network component in terms of guaranteeing obstetric beds and regulating beds, much still needs to be invested to ensure, above all, dignified and safe transportation for women in labor. In this context, the literature points out that the long distances traveled and, especially, the precarious means of public transportation, consisting of uncomfortable and often overcrowded boats, force pregnant women to travel in inadequate conditions, configuring a reality that must be changed by public management in order to implement sanitary transportation^(41)^.

This reality helps to explain the fact that, to a large extent, women in this region constitute a vulnerable population, given their social status as riverside women. Thus, in addition to facing the logistical and operational challenges of their daily lives, governed by the dynamics of the rivers and the imposition of the forests, they also face limitations due to their gender condition, considering the biopsychosocial needs that result from the processes of pregnancy, childbirth and becoming a mother in the Amazon context^(42,43)^.

Additionally, at the beginning of the COVID-19 pandemic, Brazil experienced an unprecedented crisis in the health sector, evidenced by the lack of beds and oxygen in hospitals in several states of the federation. In this scenario, there was a reduction in the number of obstetric beds, attributed to the need for intensive care beds to care for people with COVID-19^(44)^, as occurred in other countries, such as Australia and Canada^(45)^.

In this study, there was an increase in the number of cesarean sections in 2020 when compared to 2019, a phenomenon possibly attributed to the pandemic. As evidenced in the literature, fearing exposure to COVID-19, many women postponed appointments at healthcare services or faced difficulties in accessing them, and others opted for this type of delivery in advance. We must also not forget the issues related to the impossibility of having a companion during labor and the reduction in the number of professionals working in healthcare services, due to the viral infection, constituting factors that may have influenced the choice of cesarean section^(46)^.

The health crisis has worsened existing problems in the provision of these services and in access by human groups, a context in which pregnant and postpartum women were particularly affected by the interruption of prenatal consultations and the reduction of obstetric beds, contributing to an increase in maternal deaths and hindering the achievement of the goals proposed by the SDGs^(44,47)^. Furthermore, the combination of the effects of the health crisis with locoregional and socioeconomic inequalities, particularly among pregnant and postpartum women in riverside areas of Pará, has intensified perinatal challenges^(10)^.

In the historical series, the data show an increasing number of pregnant women and, consequently, greater care in the regional maternity hospital, possibly due to the interest and investments of municipalities in the Baixo Tocantins region in referring women in labor, culminating in the high local flow. Similarly, research carried out in the state of Rio de Janeiro demonstrated an increase in the percentage of pregnant women who attended obstetric reference centers, with significant regional differences^(48)^.

This context reinforces the need for integrated and collaborative strategies to deal with the challenges inherent in the displacement of pregnant women from riverside communities in the Healthcare Networks. Thus, many management actions are necessary to make this displacement viable, aiming to obtain safe and quality care for the mother/child dyad as well as better structure in the most distant municipalities. Although the Stork Network exists, which provides guarantees for prenatal, labor, childbirth, postpartum and comprehensive healthcare for children, the multiple vulnerabilities of this specific population imply accentuated risks for the dyad.

### Study limitations

The limitations of this study correspond to two factors inherent to its nature, such as the use of secondary data, therefore, subject to underreporting, and the fact that it was developed in only one integration region of the state of Pará.

### Contributions to nursing, health or public policy

By analyzing the flow of women in labor to the maternity ward, associated with the availability of obstetric beds in the Baixo Tocantins region, this study contributes to healthcare professionals and public managers, offering a different and focused look at the weaknesses of municipalities that have a lower supply of obstetric hospital care, aiming to strengthen healthcare and enhance the achievement of the SDGs, especially the third, inherent to human groups’ health.

In view of this, the territorialization, agreements among municipalities, structure and functions related to the set of care strategies highlight the importance of studies like this, especially for managers, as it allows critical reflection on the need to provide opportunities and manage changes in the territory, understanding the singularities of the region. This attitude can contribute to achieving objectives and goals in health indicators, as established by municipal, state and federal governments, through health departments and the Ministry of Health.

## CONCLUSIONS

The increase in the number of childbirths performed in the regional maternity hospital was evident, and the trend in cesarean sections followed this increase throughout the study period. Furthermore, it was observed that the social profile of childbirths followed the profile of the Brazilian national average and that using the Kernel density interpolator indicated the municipalities with the highest concentrations of postpartum women: Barcarena, Abaetetuba, Moju and Igarapé-Miri. Risk factors for unfavorable obstetric outcomes were identified, such as long distances traveled between certain municipalities of residence and the maternity hospital, and low supply of obstetric beds.

In order to promote safe childbirth, it is necessary to guarantee women’s rights to access qualified care. In this regard, the availability of obstetric beds and trained professionals in the various municipalities is a necessity, and investment should be made in the implementation and/or qualification of obstetric services in geographically strategic municipalities to avoid pilgrimage of women.

Local health managers are responsible for facilitating the safe and timely transportation of women from their municipality of residence to the regional maternity hospital. Thus, it is important to urgently discuss and implement a health transport system, since the only option available in municipalities is, as a rule, transportation for treatment outside the home and, on the islands, watercraft (boats), equipped to care for and transport the sick, injured and other people in emergency situations, not always promptly, due to the distances that prolong the journeys.

It is important to note that it is necessary to comply with the Stork Network guidelines on linking pregnant women to maternity wards, thus reassuring them, in a certain way, for childbirth. It is also necessary to invest in infrastructure in municipal healthcare institutions and in training multidisciplinary teams, as well as in carrying out other research on the subject, in order to enable or strengthen qualified care, avoid unnecessary travel and overload in regional reference services, ensuring a better response to demands in the context of obstetric health.
