# Supplementary material for: Childbirth dynamics in the riverside region of the Brazilian Amazon from the perspective of geospatialization
Source: Rev Bras Enferm. 2025 Jan 10;77(Suppl 2):e20240038. doi: 10.1590/0034-7167-2024-0038 (PMC11726818; doi:10.1590/0034-7167-2024-0038)
Supplement: Translation

# Translation: Dinâmica de partos em região ribeirinha da Amazônia brasileira na perspectiva da geoespacialização

**DOI:** 10.1590/0034-7167-2024-0038pt

**Authors:** Liandra Silva Lopes, Laura Maria Vidal Nogueira, Erlon Gabriel Rego de Andrade, Ivaneide Leal Ataíde Rodrigues, Juan Andrade Guedes, Rosinelle Janayna Coêlho Caldas, Paula Gisely Costa Silva

**Affiliations:** I Universidade Federal do Pará. Belém, Pará, Brasil; II Universidade do Estado do Pará. Belém, Pará, Brasil

**Keywords:** Análise Espacial, Epidemiologia, Obstetrícia, Parto, Saúde Pública

## Abstract

**Objetivo:**

analisar o padrão espaço-temporal de partos e o fluxo de parturientes atendidas em uma maternidade de referência regional.

**Métodos:**

estudo ecológico de 4.081 partos realizados no período de setembro/2018 a dezembro/2021 em maternidade pública na região Baixo Tocantins, Pará, Brasil. Com dados coletados em cinco fontes, construiu-se banco de dados geográficos e empregou-se análise espacial com o interpolador de densidade Kernel. Mapas foram gerados nos *softwares QGis*/3.5 e *TerraView*/4.3, calculando-se o qui-quadrado (p<0,05).

**Resultados:**

as maiores concentrações de partos, pelas vias normal e cesariana, foram evidenciadas em Barcarena (n=2.558/62,68%), Abaetetuba (n=750/18,38%), Moju (n=363/8,89%) e Igarapé-Miri (n=219/5,37%). Entre os municípios da região, dez dispunham de leitos obstétricos, totalizando 210 leitos. Nesse cenário, as parturientes percorriam até 288 km para chegar à maternidade.

**Conclusões:**

longas distâncias entre certos municípios de residência e a maternidade, e a baixa oferta de leitos obstétricos, foram identificadas como fatores de risco para desfechos obstétricos desfavoráveis.

## INTRODUÇÃO

Entre os 17 Objetivos de Desenvolvimento Sustentável (ODS), o terceiro remete à necessidade de assegurar vida saudável às populações e promover o bem-estar para todos, incorporando a meta de reduzir a taxa de mortalidade materna e acabar com as mortes evitáveis, inclusive de recém-nascidos. Trata-se de um pacto global, com metas estabelecidas para o ano de 2030, suscitando vários desafios locorregionais que implicam o alcance dos objetivos^(1)^.

No campo específico da assistência ao parto, muitos esforços estão sendo envidados nos territórios nacional e internacional, com inovação de políticas públicas, visando corresponder à propositura dos ODS. Em 2018, importante estratégia foi elaborada pelo Fundo das Nações Unidas para a Infância (UNICEF), denominada Campanha *Every Child Alive*, que incentiva a prevenção e o tratamento oportuno de complicações advindas do parto, estabelecendo duas prioridades: a qualificação dos profissionais de saúde; e a garantia de insumos necessários, em todas as instituições que prestam assistência obstétrica, para propiciar o atendimento seguro e eficaz^(2,3)^.

Nesse contexto, o Brasil tem apresentado várias inciativas para alcançar, até 2030, a meta de preservar a saúde e integridade de mulheres por ocasião do parto e não ultrapassar 70 mortes maternas por 100.000 nascidos vivos^(4)^. Entre elas, destaca-se a iniciativa da Comissão Nacional de Incorporação de Tecnologias (CONITEC) do Sistema Único de Saúde (SUS), que, em 2017, publicou as Diretrizes Nacionais de Assistência ao Parto Normal, com o intuito de padronizar as práticas obstétricas baseadas em evidências científicas, reduzir o número de intervenções desnecessárias e, assim, contribuir para promover assistência mais qualificada à mulher^(5)^.

Destacam-se, ainda, a implementação das Redes de Atenção à Saúde, como a Rede Cegonha, criada em 2011^(6)^, e o Projeto Parto Adequado, instituído em 2015^(7)^. Ressalta-se que, da concepção ao puerpério, os direitos da mulher são assegurados pela Portaria nº 1.067, de 4 de julho de 2005, que instituiu a Política Nacional de Atenção Obstétrica e Neonatal, orientando as secretarias de saúde dos municípios, dos estados e do Distrito Federal para desenvolver ações de promoção da saúde, prevenção do adoecimento e assistência às gestantes e aos recém-nascidos^(8)^.

Tais iniciativas visam reduzir a mortalidade materna, que, em 2022, no território brasileiro, alcançou cifras de 66.862 mortes na faixa etária de 10 a 49 anos. Por sua vez, no estado do Pará, considerando essa faixa etária e o mesmo ano, foram 2.873 mortes^(9)^. Entre os fatores que se relacionam com as elevadas taxas de mortalidade, estão as complicações por ocasião do parto.

Nesse contexto, é oportuno salientar que a pandemia de COVID-19 introduziu desafios adicionais na busca pela redução da mortalidade materna. Inicialmente, gestantes e puérperas não foram incluídas como grupos com maior vulnerabilidade para complicações severas da COVID-19, atrasando o início da vacinação contra a doença nesses grupos. As repercussões da pandemia foram profundas no SUS, contribuindo para desfechos perinatais desfavoráveis, como o aumento de mortes maternas por causas obstétricas indiretas e evitáveis^(10)^.

Eventos como complicações e óbitos não ocorrem de forma homogênea no território, sendo importante estudar diferenças regionais para adotar ações estratégicas direcionadas. Em que pese a legitimação das políticas públicas, ainda persistem muitos desafios para efetivá-las plenamente, devido às adversidades que se apresentam no cotidiano da atenção à saúde e da gestão dos serviços públicos. Entre os desafios, destaca-se a composição heterogênea da população brasileira, com grupos sociais exibindo taxas de mortalidade distintas, além das características geográficas do território que, em muitas realidades, comprometem o acesso aos serviços e à assistência qualificada, como ocorre nos municípios ribeirinhos da Amazônia paraense^(11)^.

Situados às margens de rios e igarapés, esses municípios enfrentam carência na oferta de serviços de saúde especializados, de maternidades e de transportes sanitários que assegurem à mulher deslocamento seguro até a unidade de saúde para realizar as consultas de pré-natal e até o local do parto, inviabilizando uma importante recomendação da Rede Cegonha. Dessa forma, visualiza-se um contraste entre as políticas públicas e a sua real aplicabilidade, sobretudo para mulheres ribeirinhas da Amazônia paraense^(12,13)^.

Ainda no contexto das políticas para atender à mulher por ocasião do parto, ressalta-se a estratégia de pactuação de serviços entre municípios, prevista como modelo no SUS, por meio do Contrato Organizativo de Ação Pública da Saúde (COAP), que norteia a organização e integração das ações e dos serviços de saúde nas regiões brasileiras, visando à integralidade da assistência e enfatizando as responsabilidades dos gestores^(14)^.

Assim, a pactuação intermunicipal tem sido adotada na gestão em saúde, contudo, há que se questionar sua aplicabilidade e adequabilidade às diversas realidades brasileiras, como no caso da região Baixo Tocantins, uma das regiões de integração do estado do Pará, que dispõe de uma maternidade de referência para atender aos 11 municípios que a compõem. Por se tratar de uma região com população ribeirinha, situações peculiares se apresentam, devendo esse modelo de pactuação de serviços entre municípios ser avaliado e monitorado regularmente.

## OBJETIVO

Analisar o padrão espaço-temporal de partos e o fluxo de parturientes atendidas em uma maternidade de referência regional.

## MÉTODOS

### Aspectos éticos

Atendeu-se à Resolução nº 466/2012 do Conselho Nacional de Saúde/Ministério da Saúde, obtendo-se autorização institucional da Secretaria de Estado de Saúde Pública do Pará (SESPA) e de uma maternidade de referência, vinculada ao SUS, com aprovação pelo Comitê de Ética em Pesquisa do Curso de Graduação em Enfermagem da Universidade do Estado do Pará. Tratando-se de pesquisa com dados secundários, utilizaram-se Termos de Autorização de Acesso aos Bancos de Dados, os quais foram assinados por representantes da SESPA e da maternidade, razão pela qual dispensou-se o consentimento de participantes.

### Desenho e local do estudo

Optou-se por estudo ecológico, empregando ferramentas da geotecnologia aplicadas à saúde. Sua redação foi guiada pelo instrumento *STrengthening the Reporting of OBservational studies in Epidemiology* (STROBE)^(15)^.

O cenário foi a região Baixo Tocantins, estado do Pará, na Amazônia brasileira, que apresenta 839.022 habitantes^(16)^, distribuídos em 11 municípios: Abaetetuba, Acará, Baião, Barcarena, Cametá, Igarapé-Miri, Limoeiro do Ajuru, Mocajuba, Moju, Oeiras do Pará e Tailândia^(17)^. É no município de Barcarena que se localiza a maternidade pública, cenário deste estudo, que recebe demanda de todos os municípios da região.

### Período, população e critérios de seleção

A maternidade iniciou suas atividades em setembro de 2018 e, em 2022, recebeu, do Ministério da Saúde, a certificação de Hospital Amigo da Criança, em reconhecimento às boas práticas, à assistência humanizada e ao incentivo à amamentação^(18)^. Em vista disso, este estudo considerou o período de setembro de 2018 a dezembro de 2021, no qual foram realizados 4.081 partos, correspondendo à totalidade inserida neste estudo. Foram incluídos os registros dos partos, cujas parturientes residiam nos municípios da região Baixo Tocantins, sem exclusão de casos.

### Protocolo do estudo

Os dados foram obtidos em agosto de 2022, com origem em cinco fontes: 1) Sistema de Informação sobre Nascidos Vivos (SINASC), cujos dados foram obtidos na SESPA e se referem ao número de nascidos vivos na maternidade, correspondente às mulheres da região Baixo Tocantins; 2) Sistema de Informações Hospitalares (SIH-SUS), com dados obtidos na Secretaria Municipal de Saúde de Barcarena (SEMUSB), extraindo-se as variáveis ano de notificação do parto, idade da mãe, cor/raça, escolaridade em anos, estado civil, duração da gestação, via de parto e local de residência; 3) banco de dados específico da maternidade, do qual foram extraídas informações complementares sobre o modo de deslocamento da parturiente, entre o seu domicílio e a maternidade, assim como se houve referenciamento por outro serviço ou se a procura pela maternidade foi espontânea; 4) Cadastro Nacional de Estabelecimentos de Saúde (CNES), por meio do Departamento de Informática do SUS (DATASUS), para consultar o número de leitos obstétricos dos municípios; 5) ambiente virtual do Instituto Brasileiro de Geografia e Estatística (IBGE), com acesso público, a fim de obter as variáveis demográficas (quantitativo populacional dos municípios) e cartográficas (latitude e longitude dos municípios) para construir as camadas de geoinformação dos limites dos municípios.

### Análise dos resultados e estatística

Realizou-se a depuração do banco de dados, no intuito de organizar as informações e excluir atributos desnecessários para o estudo, utilizando os *softwares Microsoft Office Excel*, versão 2019, e *TabWin*/Ministério da Saúde. Foi aplicado o teste estatístico não paramétrico do Qui-quadrado de proporções esperadas iguais (teste de aderência para modelos uniformes de probabilidade), com p<0,05, para analisar os dados referentes às variáveis idade da mãe, cor/raça, escolaridade, estado civil, duração da gestação e via de parto.

Construiu-se o banco de dados geográficos (BDGeo) e, em seguida, empregou-se a análise espacial, utilizando o interpolador de densidade Kernel, o qual gerou a expressão visual de densidade dos partos. Os mapas temáticos, com a distribuição de casos e o indicador de parto dos municípios, foram gerados por meio dos *softwares QGis*, versão 3.5, e *TerraView*, versão 4.3.

## RESULTADOS

Foram analisados 4.081 registros de partos, dos quais 15 (0,37%) ocorreram em 2018; 1.132 (27,74%), em 2019; 1.503 (36,83%), em 2020; e 1.431 (35,06%), em 2021, conforme a Figura 1A. Quanto às cirurgias cesarianas, em 2018, a taxa foi de 13,33 por 100 partos, sendo que, nos anos seguintes, esse indicador aumentou, com tendência linear de crescimento (Figura 1B).

**Figura 1:** Tendência da demanda e da taxa de cirurgias cesarianas das parturientes atendidas na maternidade regional, no período de setembro de 2018 a dezembro de 2021. Pará, Brasil, 2022

Em relação ao perfil social das parturientes, a faixa etária de 19 a 28 anos apresentou maior proporção (n=2.304; 56,46%), mas houve alto número de partos entre adolescentes (n=714; 17,50%), considerando a classificação do Estatuto da Criança e do Adolescente no Brasil^(19)^, que define a adolescência como período entre 12 e 18 anos de idade. Identificou-se que 2.716 (66,55%) eram pardas e que 1.252 (30,68%) não tinham registro. O tempo de formação escolar mais expressivo foi de oito a 11 anos (n=2.362; 57,88%), seguido de quatro a sete anos (n=1.059; 25,95%). No tocante ao estado civil, 2.000 (49,01%) mulheres viviam em união estável e 1.192 (29,21%) eram solteiras (Tabela 1).

**Tabela 1:** Perfil social e duração da gestação das parturientes atendidas na maternidade regional, no período de setembro de 2018 a dezembro de 2021. Pará, Brasil, 2022 (N=4.081)

Variáveis	n	%	Valor de *p*
Faixa etária			<0,0001
12 a 18 anos	714	17,50	
19 a 28 anos	2.304	56,46	
29 a 38 anos	957	23,45	
39 a 48 anos	106	2,60	
Cor/raça			<0,0001
Parda	2.716	66,55	
Branca	72	1,76	
Amarela	22	0,54	
Preta	19	0,47	
Ignorada	1.252	30,68	
Escolaridade			<0,0001
Nenhuma	9	0,22	
1 a 3 anos	147	3,60	
4 a 7 anos	1.059	25,95	
8 a 11 anos	2.362	57,88	
12 anos ou mais	402	9,85	
Ignorada	102	2,50	
Estado civil			<0,0001
Solteira	1.192	29,21	
Casada	816	20,00	
União estável	2.000	49,01	
Viúva	4	0,10	
Separada judicialmente	12	0,29	
Ignorada	57	1,40	
Duração da gestação			<0,0001
Menos de 22 semanas	71	1,74	
22 a 27 semanas	341	8,36	
28 a 31 semanas	1.302	31,90	
32 a 36 semanas	2.326	57,00	
37 a 41 semanas	41	1,00	

Quanto à duração da gestação, os valores mais expressivos foram de 32 a 36 (n=2.326; 57,00%) e 28 a 31 semanas (n=1.302; 31,90%). Cabe ressaltar que 71 (1,74%) apresentaram evolução gestacional com menos de 22 semanas e que apenas 41 (1,00%) cursaram com 37 a 41 (Tabela 1).

De acordo com a Tabela 2, as ocorrências de parto pelas vias normal e cesariana apresentaram, respectivamente, maiores proporções entre parturientes dos municípios de Barcarena (n=1.366/65,58%; n=1.192/59,66%) e Abaetetuba (n=347/16,66%; n=403/20,17%). Nesse contexto, os municípios de Limoeiro do Ajuru (n=4/0,19%; n=8/0,40%) e Tailândia (n=3/0,14%; n=7/0,35%) apresentaram os menores registros.

**Tabela 2:** Via de parto, segundo município de residência, das parturientes atendidas na maternidade regional, no período de setembro de 2018 a dezembro de 2021. Pará, Brasil, 2022 (N=4.081)

Município/ano	Via de parto						
	**Normal**						
	**2018**	**2019**	**2020**	**2021**	**Total**	**%**	**Valor de *p***
Abaetetuba	1	157	101	88	347	16,66	<0,0001
Acará	0	22	11	9	42	2,02	
Baião	0	7	11	5	23	1,10	
Barcarena	12	378	478	498	1.366	65,58	
Cametá	0	3	2	3	8	0,38	
Igarapé-Miri	0	29	34	42	105	5,04	
Limoeiro do Ajuru	0	0	2	2	4	0,19	
Mocajuba	0	2	4	5	11	0,53	
Moju	0	59	49	55	163	7,83	
Oeiras do Pará	0	5	2	4	11	0,53	
Tailândia	0	0	1	2	3	0,14	
Total	13	662	695	713	2.083	100,00	
	**Cesariana**						
	**2018**	**2019**	**2020**	**2021**	**Total**	**%**	**Valor de *p***
Abaetetuba	0	126	166	111	403	20,17	<0,0001
Acará	0	0	8	8	16	0,80	
Baião	0	6	8	9	23	1,15	
Barcarena	2	251	490	449	1.192	59,66	
Cametá	0	6	1	3	10	0,50	
Igarapé-Miri	0	22	44	48	114	5,71	
Limoeiro do Ajuru	0	4	2	2	8	0,40	
Mocajuba	0	1	6	9	16	0,80	
Moju	0	52	78	70	200	10,01	
Oeiras do Pará	0	2	4	3	9	0,45	
Tailândia	0	0	1	6	7	0,35	
Total	2	470	808	718	1.998	100,00	

Considerando o somatório de partos pelas vias normal e cesariana, a Figura 2 ilustra as diferentes concentrações entre os municípios. Assim, as maiores concentrações foram evidenciadas em Barcarena (n=2.558; 62,68%), Abaetetuba (n=750; 18,38%), Moju (n=363; 8,89%) e Igarapé-Miri (n=219; 5,37%), e as menores concentrações foram registradas nos outros municípios: Acará (n=58; 1,42%), Baião (n=46; 1,13%), Mocajuba (n=27; 0,66%), Oeiras do Pará (n=20; 0,49%), Cametá (n=18; 0,44%), Limoeiro do Ajuru (n=12; 0,29%) e Tailândia (n=10; 0,25%).

**Figura 2:** Distribuição espacial dos partos realizados na maternidade regional, no período de setembro de 2018 a dezembro de 2021. Pará, Brasil, 2022

Segundo a Figura 3A, entre os 11 municípios que constituem a região Baixo Tocantins, dez dispunham de leitos obstétricos informados no CNES, totalizando 210 leitos, assim distribuídos: Barcarena (65 leitos), Abaetetuba (60 leitos), Cametá (42 leitos), Moju (18 leitos), Igarapé-Miri (dez leitos), Limoeiro do Ajuru (cinco leitos), Baião (três leitos), Oeiras do Pará (três leitos), Acará (dois leitos) e Mocajuba (dois leitos).

**Figura 3:** Distribuição espacial dos leitos obstétricos e do fluxo de parturientes atendidas na maternidade regional, no período de setembro de 2018 a dezembro de 2021. Pará, Brasil, 2022

Por sua vez, a Figura 3B contempla a mobilidade das parturientes em busca de assistência hospitalar para parir, com fluxos de até 288 km entre o município de residência e a maternidade regional. Os municípios de Baião, Oeiras do Pará e Tailândia são os mais distantes em relação à maternidade, cenário em que o acesso de Oeiras do Pará para Barcarena, onde está localizada essa instituição, é exclusivamente realizado por via fluvial, devido à existência de rios que entrecortam o território. Embora estejam relativamente próximos de Barcarena, os municípios de Limoeiro do Ajuru e Cametá são separados por rios, representando importante obstáculo para o deslocamento.

## DISCUSSÃO

O padrão espacial dos partos indica predominância de mulheres oriundas do município onde está localizada a maternidade regional e de municípios mais próximos geograficamente. Entretanto, é possível visualizar importante fluxo em toda a região Baixo Tocantins.

É fato que a demanda de partos nessa maternidade está relacionada, ainda, ao contingente populacional e à distância geográfica, que implica facilidade ou dificuldade de acesso quanto à logística de deslocamento. Dessa forma, a maior proporção evidenciada no município de Barcarena deve-se ao fato de constituir a sede da maternidade, e, em Abaetetuba, a proporção é explicada por ser município vizinho, com maior facilidade para deslocamento intermunicipal.

A assistência à mulher, por ocasião do parto, apresentou tendência de crescimento no período estudado, e as cesarianas acompanharam essa tendência. Compreende-se que, pelo fato de ser a primeira maternidade de referência na região, as mulheres passaram a optar por ela ou foram referenciadas em virtude da possível oferta de assistência mais qualificada, ou mesmo pela escassez de leitos obstétricos nos diversos municípios, além da expectativa de serem melhor atendidas.

Entende-se que a demanda crescente está vinculada à divulgação da implantação do serviço, promovendo a busca espontânea pelas mulheres e o referenciamento pelas equipes de saúde nos municípios da região. A característica dessa instituição como maternidade de referência implica a recepção de casos com maior complexidade, os quais necessitam de atenção diferenciada, tais como casos que demandam cirurgia cesariana^(20-21)^. Mesmo assim, a política do hospital valoriza a viabilização do parto normal, sempre que indicado, repercutindo na redução da taxa de cesarianas em 2021, quando comparada à de 2020.

A realização de cirurgias cesarianas, sem a devida indicação, vem sendo pauta de debates e pesquisas, no intuito de propiciar a sua redução, considerando que as taxas desse tipo de cirurgia aumentaram globalmente, correspondendo a mais de um para cada cinco partos (21,00%), segundo dados da Organização Mundial da Saúde (OMS) e da Organização Pan-Americana da Saúde (OPAS). Na América Latina, as cesarianas totalizam quatro de cada dez partos, e, no Brasil, já superaram o número de partos normais, apesar de o acesso à cirurgia ser desigual entre as mulheres^(22)^.

Achados semelhantes foram obtidos em estudo nacional, que investigou as modalidades de parto no SUS, identificando aumento de cesarianas em todas as regiões do país nos últimos anos. Não obstante, a região Norte se destacou pela predominância de partos normais, mas tem apresentado crescimento exponencial de cesarianas, indicando que poderá acompanhar o índice das demais regiões do território brasileiro nos próximos anos^(23)^. No contexto dos planos de saúde privados no Brasil, essa tendência de crescimento é expressiva, visto que, em 2019, dos 287.166 partos realizados, 84,76% foram por via cesariana^(24)^.

Apesar de a cirurgia cesariana demandar plausível indicação clínica para ser realizada, o atual cenário brasileiro sugere o aumento do número de procedimentos desnecessários e potencialmente prejudiciais para o binômio mãe/filho^(25)^. Todavia, salienta-se que a maternidade regional de Barcarena atende, além das parturientes de risco habitual, as de alto risco, sendo justificável o quantitativo de cesarianas nela realizadas^(26)^.

Quanto ao perfil social das parturientes, a faixa etária prevalente acompanha a média nacional, com idade entre 19 e 28 anos^(23)^. Não obstante, a região é palco de gravidez entre adolescentes, demandando políticas intersetoriais mais arrojadas. Para reduzir a incidência de gravidez precoce e/ou indesejada, destaca-se a estratégia de Aprimoramento e Inovação no Cuidado e Ensino em Obstetrícia e Neonatologia (APICE ON), proposta pelo Ministério da Saúde, em parceria com a Empresa Brasileira de Serviços Hospitalares (EBSERH), a Associação Brasileira de Hospitais Universitários de Ensino (ABRAHUE), o Ministério da Educação (MEC) do Brasil, o Instituto Fernandes Figueira (IFF) e a Fundação Oswaldo Cruz (FIOCRUZ). Entre outras finalidades, essa estratégia visa alcançar as melhores práticas em planejamento reprodutivo no pós-parto, no pós-aborto e na atenção às mulheres vítimas de violência sexual e/ou que evoluíram com abortamento ou realizaram aborto legal^(27)^.

No Brasil, em 2020, a cada 1.000 adolescentes, 53 engravidaram precocemente, de acordo com o Relatório sobre a Situação da População Mundial, divulgado pelo Fundo de População das Nações Unidas (UNFPA), apontando que, no país, esse valor está acima do indicador mundial, que é de 41 casos a cada 1.000 adolescentes. Dessa maneira, o UNFPA reforça a importância da saúde sexual e reprodutiva e de incentivá-la desde a adolescência, em todos os contextos socioeducacionais, além do acesso às informações confiáveis^(28)^.

Ainda sobre o perfil das parturientes, a predominância de mulheres pardas reflete o cenário étnico do país, cujo estudo nacional de bases populacionais, relativo ao período de 1930 a 2018, identificou 61,54% de parturientes pardas^(29)^. Na população brasileira, a prevalência das raças parda e preta é resultado da miscigenação entre índios, africanos e demais etnias que participaram da colonização do país, o qual apresenta a maior miscigenação do mundo, em virtude da descendência heterogênea^(30)^.

A escolaridade, com tempo de formação igual ou superior a oito anos, representa aspecto positivo, pois implica a possibilidade de reduzir fatores de risco na gravidez, no parto e no puerpério. Esse entendimento se ancora no fato de que a escolaridade inferior a oito anos limita, de diferentes maneiras, o desenvolvimento satisfatório dos cuidados na atenção pré-natal. Além disso, a baixa instrução da mãe figura como critério de elegibilidade para a classificação de recém-nascido de risco ao nascer^(31)^.

A Pesquisa Nacional por Amostra de Domicílios Contínua (PNAD/IBGE), em 2021, apontou que a média dos anos de estudo da população brasileira com 18 a 29 anos, no período de 2012 a 2020, foi de 11,4 anos. Nesse contexto, é interessante notar que o Brasil exibe tendência de escolaridade com oito anos ou mais, entretanto as regiões Norte e Nordeste ainda apresentam as menores médias de anos de formação escolar no país, com 11,2 e 11,1 anos, respectivamente^(32)^. O aumento dos anos estudados pela população brasileira se tornou possível com a redução das desigualdades entre os grupos humanos, apesar de ainda se encontrar muitas desigualdades sociais no país^(33)^.

Quanto à situação conjugal, a maioria vivia em união estável, semelhante ao resultado de outro estudo, que demonstrou a média nacional de 51,20% de mulheres vivendo com parceiro no momento do parto^(23)^. Isso difere dos achados de pesquisa realizada no município do Rio de Janeiro, na qual as mães solteiras totalizaram 62,60%^(29)^.

A maioria das parturientes não completou o período ideal de gestação, pois apresentou idade gestacional inferior a 37 semanas. Sabe-se que a prematuridade é uma das principais causas de óbito no período neonatal (primeiros 28 dias de vida após o parto) e, neste estudo, o período em que mais se evidenciou partos prematuros foi de 32 a 36 semanas, seguindo a média nacional de 79,00% de partos prematuros^(34)^. Assim, considerando, aproximadamente, 3.000.000 de nascimentos por ano, em todo o território brasileiro, 2.370.000 ocorrem com idade gestacional inferior a 37 semanas^(35)^.

Considerando as parturientes nos contextos social e econômico da região, visualizam-se diferenças nos Índices de Desenvolvimento Humano Municipal (IDHM) e na renda *per capita* entre os municípios, repercutindo na qualidade de vida e na exposição a riscos maternos. O IDHM compõe três importantes indicadores de desenvolvimento humano: longevidade, educação e renda. Portanto, o nível medido expressa as condições sociais e financeiras para nascer e crescer^(36)^.

No cenário estudado, Barcarena apresenta o maior IDHM (0,662), seguido por Abaetetuba e Baião, com 0,628 e 0,578, respectivamente. Por sua vez, o menor IDHM corresponde ao município de Acará (0,506). Destaca-se, também, que Barcarena concentra a maior renda *per capita* em reais (R$ 47.684,37), seguido por Acará e Limoeiro do Ajuru, com R$ 11.892,64 e R$ 14.480,24, respectivamente. O município com menor renda é Cametá, com R$ 7.271,26^(36)^.

A influência do IDHM foi evidenciada em estudo realizado na região da Serra Catarinense, Brasil, cujos resultados indicaram associação com melhores condições de vida do recém-nascido e maior escolaridade materna, corroborando a influência dos fatores socioeconômicos no estado de vida e de saúde materno e neonatal^(37)^. Ainda nessa perspectiva, estudos regionais mostraram que menores valores de IDHM estão associados à maior incidência de gravidez na adolescência^(38,39)^.

Municípios com menores rendas *per capita* apresentaram menor disponibilidade de leitos obstétricos, com exceção do município de Cametá. No Brasil, a oferta de leitos hospitalares é de 2,3 para cada 1.000 habitantes, inferior à média global, 3,2 por 1.000 habitantes, e ao número previamente recomendado pelo Ministério da Saúde, de 2,5 a 3,0 por 1.000 habitantes^(40)^. Nesse sentido, é importante inferir que as desigualdades sociais, evidenciadas por baixa renda *per capita*, aumentam os riscos para gravidez precoce e impactam negativamente os períodos prée pós-natal.

Obedecendo ao que é preconizado pelo SUS, ressalta-se que a maternidade atende demanda de todos os municípios da região e, apesar de assegurar o componente da Rede Cegonha no que tange à garantia de leitos obstétricos e à regulação de leitos, muito ainda precisa ser investido para garantir, sobretudo, deslocamento digno e seguro às parturientes. Nesse contexto, a literatura aponta que as longas distâncias percorridas e, especialmente, o meio de transporte coletivo precário, constituído por embarcações pouco confortáveis, muitas vezes superlotadas, obrigam as gestantes a viajar em condições inadequadas, configurando realidade que deve ser modificada pela gestão pública, a fim de implementar transporte sanitário^(41)^.

Essa realidade contribui para explicar o fato de que, em grande parte, as mulheres dessa região constituem uma população vulnerável, tendo em vista sua condição social como mulheres ribeirinhas. Dessa maneira, além de enfrentarem os desafios logísticos e operacionais do cotidiano que vivenciam, regido pela dinâmica dos rios e pela imposição das florestas, também enfrentam limitações por sua condição de gênero, diante das necessidades biopsicossociais que resultam dos processos de gestar, parir e tornar-se mãe no contexto amazônico^(42,43)^.

Adicionalmente, no início da pandemia de COVID-19, o Brasil experimentou uma crise sem precedentes no setor de saúde, evidenciada pela carência de leitos e oxigênio em hospitais de vários estados da federação. Nesse cenário, houve redução no número de leitos obstétricos, atribuída à necessidade de leitos de terapia intensiva para atender pessoas com COVID-19^(44)^, a exemplo do que ocorreu em outros países, como Austrália e Canadá^(45)^.

Neste estudo, houve aumento no número de cesarianas em 2020, quando comparado a 2019, fenômeno possivelmente atribuído à pandemia. Como evidenciado na literatura, temendo a exposição à COVID-19, muitas mulheres adiaram consultas em serviços de saúde ou enfrentaram dificuldades de acesso, e outras optaram, previamente, por essa via de parto. Não se pode esquecer, também, das questões relacionadas à impossibilidade de ter um acompanhante durante o parto e à redução do número de profissionais que atuavam nos serviços de saúde, devido à infecção viral, constituindo fatores que podem ter influenciado a escolha pela cesariana^(46)^.

A crise sanitária agravou problemas já existentes na oferta desses serviços e no acesso dos grupos humanos, contexto em que gestantes e puérperas foram especialmente afetadas pela interrupção de consultas pré-natais e redução de leitos obstétricos, contribuindo para elevar as mortes maternas e obstaculizar o alcance das metas propostas pelos ODS^(44,47)^. Além disso, a combinação dos efeitos da crise sanitária com as desigualdades locorregional e socioeconômica, particularmente entre gestantes e puérperas de áreas ribeirinhas do Pará, intensificou os desafios perinatais^(10)^.

Na série histórica, os dados evidenciam número crescente de gestantes e, por consequência, maior atendimento na maternidade regional, possivelmente devido ao interesse e aos investimentos dos municípios da região Baixo Tocantins para encaminhar parturientes, culminando no elevado fluxo local. De maneira semelhante, pesquisa realizada no estado do Rio de Janeiro demonstrou aumento do percentual de gestantes que realizaram itinerários em polos de referência obstétrica, com importantes diferenças regionais^(48)^.

Esse contexto reforça a necessidade de estratégias integradas e colaborativas para lidar com os desafios inerentes ao deslocamento de gestantes ribeirinhas nas Redes de Atenção à Saúde. Assim, muitas ações de gestão são necessárias para viabilizar esse deslocamento, visando obter assistência segura e de qualidade para o binômio mãe/filho, bem como melhor estrutura dos municípios mais distantes. Embora exista a Rede Cegonha, que dispõe sobre as garantias de pré-natal, parto, nascimento, puerpério e atenção integral à saúde da criança, as múltiplas vulnerabilidades dessa população específica implicam riscos acentuados para o binômio.

### Limitações do estudo

As limitações deste estudo correspondem a dois fatores inerentes à sua natureza: utilização de dados secundários, portanto, passíveis de subnotificação; e o fato de ter se desenvolvido em apenas uma região de integração do estado do Pará.

### Contribuições para as áreas de enfermagem, saúde ou políticas públicas

Com a análise do fluxo de parturientes para a maternidade, associada à disponibilidade de leitos obstétricos na região Baixo Tocantins, este estudo contribui com profissionais de saúde e gestores públicos, oferecendo um olhar diferenciado e enfocado nas fragilidades dos municípios que apresentam menor oferta de assistência hospitalar obstétrica, visando fortalecer a atenção à saúde e potencializar o alcance dos ODS, especialmente o terceiro, inerente à saúde dos grupos humanos.

Em vista disso, a territorialização, as pactuações entre municípios, a estrutura e as funções relativas ao conjunto das estratégias de atenção evidenciam a importância de estudos como este, sobretudo aos gestores, pois permite refletir criticamente sobre a necessidade de oportunizar e administrar mudanças no território, compreendendo as singularidades da região. Essa atitude pode contribuir para alcançar objetivos e metas nos indicadores de saúde, conforme estabelecido pelos governos municipais, estadual e federal, por meio das secretarias de saúde e do Ministério da Saúde.

## CONCLUSÕES

Evidenciou-se o aumento do número de partos realizados na maternidade regional, e a tendência de cirurgias cesarianas acompanhou esse aumento ao longo do período estudado. Além disso, observou-se que o perfil social dos partos acompanhou o perfil da média nacional brasileira e que o uso do interpolador de densidade Kernel apontou os municípios com maiores concentrações de parturientes: Barcarena, Abaetetuba, Moju e Igarapé-Miri. Foram identificados fatores de risco para desfechos obstétricos desfavoráveis, como longas distâncias percorridas entre certos municípios de residência e a maternidade, e baixa oferta de leitos obstétricos.

Com o intuito de promover o nascimento seguro, é preciso garantir os direitos da mulher ao acesso à assistência qualificada. Nesse sentido, a disponibilidade de leitos obstétricos e de profissionais capacitados nos diversos municípios figura como necessidade, devendo-se investir na implantação e/ou qualificação de serviços obstétricos em municípios geograficamente estratégicos para evitar a peregrinação das mulheres.

Aos gestores de saúde locais, confere-se a responsabilidade de viabilizar o deslocamento da mulher, de forma segura e oportuna, do seu município de residência até a maternidade regional. Dessa maneira, ressalta-se a urgência de discutir e implantar um sistema de transporte sanitário, visto que a possibilidade de oferta dos municípios ainda é, via de regra, o transporte para tratamento fora de domicílio e, nas ilhas, a viatura aquática (lancha), equipada para atender e transportar doentes, feridos e outras pessoas em situação de urgência/emergência, nem sempre com prontidão, em virtude das distâncias que prolongam os deslocamentos.

Salienta-se que é preciso cumprir as orientações da Rede Cegonha sobre vincular as gestantes à maternidade, tranquilizando-as, de certa maneira, para o parto. Também é necessário investir em infraestrutura nas instituições de saúde dos municípios e na capacitação das equipes multiprofissionais, bem como na realização de outras pesquisas sobre o tema, a fim de possibilitar ou fortalecer o atendimento qualificado, evitar deslocamentos desnecessários e sobrecarga nos serviços de referência regional, garantindo melhor resposta às demandas no contexto da saúde obstétrica.
